# Maternal and paternal depression and child mental health trajectories: evidence from the Avon Longitudinal Study of Parents and Children

**DOI:** 10.1192/bjo.2021.959

**Published:** 2021-09-24

**Authors:** Priya Rajyaguru, Alex S. F. Kwong, Elizabeth Braithwaite, Rebecca M. Pearson

**Affiliations:** Centre for Academic Mental Health, University of Bristol, Bristol, and Oxford Health NHS Foundation Trust, UK; Population Health Sciences, Bristol Medical School, and Medical Research Council Integrative Epidemiology Unit, University of Bristol, Bristol, UK; Department of Psychology, School of Health, Psychology and Social Care, Manchester Metropolitan University, Manchester, UK; Population Health Sciences, Bristol Medical School, and Medical Research Council Integrative Epidemiology Unit, University of Bristol, and National Institute for Health Research Bristol Biomedical Research Centre, Bristol, UK

**Keywords:** ALSPAC, child depression, adolescent depression, maternal depression, paternal depression, trajectories

## Abstract

**Background:**

The relationships between offspring depression profiles across adolescence and different timings of parental depression during the perinatal period remain unknown.

**Aims:**

To explore different timings of maternal and paternal perinatal depression in relation to patterns of change in offspring depressive mood over a 14 year period.

**Method:**

Data were obtained from the Avon Longitudinal Study of Parents and Children (ALSPAC). Parental antenatal depression (ANTD) was assessed at 18 weeks gestation, and postnatal depression (PNTD) at 8 weeks postpartum. Population-averaged trajectories of offspring depressive symptoms were estimated using the Short Mood and Feelings Questionnaire (SMFQ) on nine occasions between 10 and 24 years of age.

**Results:**

Full data were available for 5029 individuals. Offspring exposed to both timings of maternal depression had higher depressive symptoms across adolescence compared with offspring not exposed to ANTD or PNTD, characterised by higher depressive symptoms at age 16 (7.07 SMFQ points (95% CI = 6.19, 7.95; *P* < 0.001)) and a greater rate of linear change (0.698 SMFQ points (95% CI = 0.47, 0.93; *P* = 0.002)). Isolated maternal ANTD and to a lesser extent PNTD were also both associated with higher depressive symptoms at age 16, yet isolated maternal PNTD showed greater evidence for an increased rate of linear change across adolescence. A similar pattern was observed for paternal ANTD and PNTD, although effect sizes were attenuated.

**Conclusions:**

This study adds to the literature demonstrating that exposure to two timings of maternal depression (ANTD and PNTD) is strongly associated with greater offspring trajectories of depressive symptoms.

There is evidence linking maternal antenatal depression (ANTD), maternal postnatal depression (PNTD) and, more recently, paternal PNTD to a range of child outcomes including emotional, behavioural, cognitive and physical health consequences.^1^ However, the underlying mechanisms remain largely unknown. One perspective is that different timings of parental mental illness (i.e. offspring exposure before and/or after birth) may be linked to some pathways more than others in relation to child outcomes (see Stein et al^1^ for a comprehensive overview). We hypothesise that a child exposed to more timings of parental depression is at greater, cumulative risk of depression. One may further hypothesise that such children will also be more vulnerable to genetic risk load when considering the intergenerational transmission of depression. However, those exposed to maternal ANTD only may experience a specific ‘pre-programmed’ effect owing to the *in utero* environment uniquely provided by mothers. There is growing evidence that exposure to prenatal maternal stress increases risk of offspring affective problems and emotional instability.^2,3^ By contrast, exposure to PNTD in either parent may reflect risk transmitted via altered parenting behaviour, and the overall effect may be similar with exposure to each parent owing to a shared parenting pathway.^4^ In addition, given existing epidemiological evidence of gender differences^5^ and animal evidence for a female fetal vulnerability to stress hormones in relation to maternal ANTD exposure (for an overview of the animal literature, see Kapoor et al^6^), it is possible that female offspring may be more vulnerable to the effects of maternal ANTD. The unique research design presented here allows us to explore patterns of data that could indicate different mechanisms by which the intergenerational transmission of depression may occur. The key hypothesis is that in addition to high genetic risk load (presence of ANTD and PNTD in either parent or both parents) and risk transmitted via environmental means, including altered parenting behaviour (PNTD in either parent or both parents), there will be additional biological risk from exposure to maternal ANTD via prenatal programming mechanisms. In addition, female offspring exposed to maternal ANTD may be at greater risk of depression than males exposed to maternal ANTD.

Thus far the literature has focused on specific time points for child outcomes, ranging from birth up to age 18,^7^ yet depression is known to change across adolescence and adulthood, with differential risk factors associated with different patterns of depression.^8^ Recent studies using advanced methods have attempted to explore trajectories of offspring risk in relation to parental perinatal mental health (e.g. O'Donnell et al^9^). This provides an opportunity to explore the nature of offspring mental health outcomes and how this may change over different periods of development. However, such research has been limited by the number of exposure timings available (in relation to parental ANTD and PNTD), the use of parent-reported outcome measures only and the total number of outcome assessments available (e.g. O'Donnell et al,^9^ where available outcome data were limited up to age 13). Thus, the potential effects of parental perinatal depression on changes in offspring depression across childhood, adolescence and into adulthood remain unclear, as do the associations with overall levels of depression across this period. This study aimed to directly address this problem by exploring different timings of maternal and paternal depression (ANTD and PNTD, in isolation and combination) in relation to patterns of change in child, adolescent and early adult depressive mood. Using a large longitudinal cohort, we aimed to address the overall level of depression and rate of change in offspring depressive symptoms specifically.

## Method

### Study sample

This retrospective cohort study used data from the Avon Longitudinal Study of Parents and Children (ALSPAC), a birth cohort that recruited pregnant women residing in Avon, UK, with expected dates of delivery 1 April 1991 to 31 December 1992.^10–12^ The initial cohort consisted of 14 062 live births; when the oldest child participants were approximately 7 years of age, the initial sample was bolstered with participants who had failed to join the study originally. As a result, for all analyses involving data from the age of 7 onwards, there was a total of 14 901 children alive at 1 year of age. Ethical approval for the study was obtained from the ALSPAC Ethics and Law Committee and the Local Research Ethics Committees. Informed consent for the use of data collected via questionnaires and clinics was obtained from participants following the recommendations of the ALSPAC Ethics and Law Committee at the time.

The study website contains details of all the data that are available through a fully searchable data dictionary and variable search tool: http://www.bristol.ac.uk/alspac/researchers/our-data.^13^ In addition, some of the data were collected using REDCap (https://projectredcap.org/resources/citations/).^14,15^

### Measures

#### Parental depression

Symptoms of parental depression were measured using the well-established Edinburgh Postnatal Depression Scale (EPDS).^16^ The EPDS is a ten-item self-report depression questionnaire validated for use in the perinatal period because it avoids physical symptoms.^16^ It is also validated for use outside the perinatal period and in men.^17–19^ Scores of >12 have a high sensitivity and specificity (estimates vary between 0.60–0.96 for sensitivity and 0.45–0.97 for specificity^19^ in predicting clinically diagnosed major depressive disorder^18–20^). In this work, scores of >12 were used as a binary cut-off with regard to the presence of parental perinatal depressive disorder. ANTD in mothers and fathers was assessed at 18 weeks gestation, and PNTD in mothers and fathers was assessed at 8 weeks postpartum.

#### Offspring depression

Self-reported depressive symptoms were measured on nine occasions between ages 10 and 24 using the Short Mood and Feelings Questionnaire (SMFQ).^21^ The SMFQ is a 13 item questionnaire widely used in adolescents that measures the presence of depressive symptoms over the past 2 weeks. In this study, it was administered via postal questionnaire or in clinics. Each item is scored between 0 and 2, with a summed total score ranging between 0 and 26. In this work, the total summed score was used in the analyses. The SMFQ correlates strongly with clinical depression^22,23^ and has been used to explore trajectories of depressive symptoms in other studies.^8,24,25^ Descriptive information on the SMFQ can be found in Supplementary Table 1 available at https://doi.org/10.1192/bjo.2021.959.

#### Confounding variables

The following confounders were included based upon previous literature examining early social risk factors and trajectories of depressive symptoms:^8,24–26^ sex (coded as a dummy variable for being female (male = 0; female = 1)), maternal/paternal educational attainment at birth (A-level or higher versus O-level versus less than O-level), parity (first-born versus second-born versus third-born or more) and maternal/paternal smoking in pregnancy (no versus yes). Maternal analyses were adjusted for current paternal depression, and paternal analyses were adjusted for current maternal depression.

### Analysis

Trajectories of depressive symptoms were estimated using multilevel growth-curve modeling.^27^ Briefly, multilevel growth-curve models create population-averaged trajectories, with individual level trajectories varying around this population average (i.e. each person may have their own trajectory that deviates from the population average). Descriptive statistics and previous analysis of this data have shown that the change in depressive symptoms is non-linear and fluctuates over adolescence and young adulthood.^8^ To model this non-linearity, a multilevel quartic growth-curve polynomial model was chosen, in line with previous research using higher-order multilevel growth-curve polynomials for examining trajectories of depressive symptoms.^8^ Age was grand-mean centred to 16.53 years (the mean age of all assessments) in order to improve interpretation,^28^ as the model intercept and intercept variance then corresponded to the middle of adolescence. Such models provide an estimate of depressive symptom scores at a given intercept age but also quantify how depressive symptoms change over time through linear, quadratic, cubic and quartic polynomial effects (slope or rate-of-change terms). In all analyses, the polynomial age terms were allowed to vary randomly across individuals to capture each individual's unique trajectory (i.e. a random intercept and slope model). Further information regarding model selection and model equations can be found in the Supplementary material.

We ran separate models to examine the association between maternal and paternal depression and offspring depressive symptom trajectories. The first model examined population-averaged trajectories for maternal depression, which was classified into four groups: no ANTD/no PNTD (baseline group), yes ANTD/no PNTD, no ANTD/yes PNTD and yes ANTD/yes PNTD. These groups were then interacted with the intercept and four polynomial terms to create four distinct trajectories of offspring depressive symptoms corresponding to the four groups. The same method was used for the paternal analysis. We also included interaction terms for gender and stratified analyses to run exploratory analyses on whether these effects differed by offspring sex.

All analyses were conducted using Stata 15^29^ with the user-written runmlwin command,^30^ which calls the standalone multilevel modelling package MLwiN v3.01 (www.cmm.bristol.ac.uk/MLwiN/index.shtml). The Stata code used in this analysis can be found at https://github.com/kwongsiufung.

### Missing data

Missing data in the trajectories analysis were handled using full information maximum likelihood (FIML), which assumes that the data are missing at random.^31^ The FIML approach assumes that the probability of an individual missing a depressive symptom measure does not depend on their underlying depressive symptoms on that occasion, given their observed trajectory on other occasions. Previous analysis has used FIML to examine trajectories of depressive symptoms in ALSPAC and found that this method handles missing data well in trajectories analysis.^8^ To maximise power, we included individuals in this analysis if they had at least one measurement of depressive symptoms.

### Sensitivity analyses

Sensitivity analyses using a more stringent threshold (only including individuals with at least four SMFQ time point measures rather than one) yielded similar results (Supplementary Fig. 1 and Table 2). As a further check to examine the effects of parental depression on offspring trajectories, we also included a later time point of parental depression as a covariate in our models when the offspring were roughly 22 years old, yielding similar findings (Supplementary Table 3).

## Results

### Descriptive results

Table 1 provides an overview of offspring demographics as per the exposure, outcome and final sample, and those excluded for the maternal analyses. Supplementary Table 4 outlines the offspring demographics for the paternal analyses. With respect to the primary outcome measure, the SMFQ reliability coefficient was stable (α = 0.8–0.92) across the nine time points of offspring outcome assessment. For further descriptive information with regards to the SMFQ used in this study, please see Supplementary Table 1. The final analyses were composed of complete, adjusted data for 5029 offspring in the maternal analyses and 4534 offspring in the paternal analyses (Tables 2 and 4). For the unadjusted analyses, please see Supplementary Tables 5 and 6.

**Table 1:** Participant demographics for maternal depression exposure

	Sample with complete exposure data (maternal depression)^a^, *N* (%)	Sample with complete outcome data (at least one offspring depression)^b^, *N* (%)	Sample with complete exposure, outcome and confounding data^c^, *N* (%)
Sex			
Male	5369 (51.41)	4495 (47.85)	2447 (48.66)
Female	5075 (48.59)	4899 (52.15)	2582 (51.34)
Maternal education			
A Level or higher	3739 (36.94)	3453 (40.87)	2814 (44.04)
GCSE/O Level	6382 (63.06)	4996 (59.13)	2215 (55.04)
Parity			
First-born	4573 (44.51)	3918 (45.94)	2465 (49.02)
Second-born	3695 (35.96)	3041 (35.66)	1745 (34.70)
Third-born +	2006 (19.53)	1569 (18.40)	819 (16.29)
Smoking			
No prenatal smoking	8087 (77.43)	6942 (80.23)	4202 (83.56)
Yes postnatal smoking	2357 (22.57)	1711 (18.77)	827 (16.44)
Paternal depression			
No ANTD/no PNTD	6413 (94.48)	5284 (94.63)	4770 (94.85)
Yes ANTD/no PNTD	156 (2.30)	121 (2.17)	103 (2.05)
No ANTD/yes PNTD	141 (2.08)	115 (2.06)	99 (1.97)
Yes ANTD/yes PNTD	78 (1.15)	64 (1.15)	57 (1.13)

ANTD, antenatal depression; PNTD, postnatal depression; SMFQ, Short Mood and Feelings Questionnaire; N, number of offspring.

a. Number of offspring for whom data on maternal perinatal depression were available. Reading down the exposure sample column, the figures also indicate the number of offspring who had data available on each individual variable in addition to maternal depression information.

b. Number of offspring for whom outcome data of at least one SMFQ result were available. Reading down the outcome sample column, the figures also indicate the number of offspring who had data available on each individual variable in addition to having at least one SMFQ result.

c. The number of offspring for whom data were available on maternal depression, confounding variables and at least one SMFQ result.

**Table 2:** Maternal adjusted trajectories of offspring depressive symptoms (*n* = 5029)

Parameter	Beta	95% CIs		s.e.	*P*-value	Baseline + risk group score parameter	95% CI + risk group score parameter	
No ANTD/no PNTD Intercept	5.353	5.144	5.562	0.107	<0.001	–	–	–
No ANTD/no PNTD age^a^	0.328	0.290	0.366	0.020	<0.001	–	–	–
No ANTD/no PNTD age^2^b^	−0.088	−0.099	−0.076	0.006	<0.001	–	–	–
No ANTD/no PNTD age^3^c^	−0.005	−0.006	−0.004	0.001	<0.001	–	–	–
No ANTD/no PNTD age^4^d^	0.002	0.001	0.002	0.0001	<0.001	–	–	–
Yes ANTD/no PNTD intercept	1.430	0.868	1.992	0.287	<0.001	6.783	6.219	7.347
Yes ANTD/no PNTD age^a^	0.092	−0.052	0.235	0.073	0.21	0.420	0.282	0.558
Yes ANTD/no PNTD age^2^b^	−0.046	−0.091	−0.001	0.023	0.046	−0.133	−0.177	−0.090
Yes ANTTD/no PNTD age^3^c^	−0.001	−0.005	0.003	0.002	0.593	−0.006	−0.009	0.002
Yes ANTD/no PNTD age^4^d^	0.001	−0.00001	0.002	0.0005	0.054	0.002	0.00200	0.003
No ANTD/yes PNTD intercept	0.776	0.114	1.439	0.338	0.022	6.130	5.466	6.793
No ANTD/yes PNTD age^a^	0.179	0.010	0.348	0.086	0.038	0.507	0.342	0.672
No ANTD/yes PNTD age^2^b^	0.002	−0.051	0.054	0.027	0.953	−0.086	−0.137	−0.035
No ANTD/yes PNTD age^3^c^	−0.002	−0.006	0.003	0.002	0.412	−0.007	−0.011	−0.002
No ANTD/yes PNTD age^4^d^	0.0000	−0.001	0.001	0.001	0.989	0.002	0.001	0.003
Yes ANTD/yes PNTD intercept	1.719	0.844	2.594	0.447	<0.001	7.072	6.191	7.953
Yes ANTD/yes PNTD age^a^	0.370	0.140	0.601	0.117	0.002	0.698	0.471	0.925
Yes ANTD/yes PNTD age^2^b^	−0.035	−0.108	0.038	0.037	0.348	−0.122	−0.194	−0.051
Yes ANTD/yes PNTD age^3^c^	−0.008	−0.014	−0.002	0.003	0.01	−0.013	−0.018	−0.007
Yes ANTD/yes PNTD age^4^d^	0.001	−0.0003	0.003	0.001	0.124	0.003	0.0010	0.004

The No ANTD/no PNTD variable is the baseline (reference) group. There are four groups. Each group has an intercept and four terms resulting in four trajectories per group. The different terms (a–d) for each group are further parameters to account for the non-linearity of the trajectories as seen by the fact that the slope is steeper at different ages. For further information on the nature of change in rate of depressive symptoms as per the four terms at different ages, please see the Supplementary material. The intercept for each group was determined by manually adding the baseline intercept for No ANTD/no PNTD to the intercept of the group being compared. Similarly, the rate of change for each subsequent group was determined by adding the rate of change for the baseline group to the rate of change of the group being compared. This is highlighted in the column entitled ‘Baseline + risk group score parameter’. For ease of interpretation, we present the original regression coefficients and their 95% CI, s.e. and *P*-values first. The s.e. and *P*-value represent the difference between the baseline trajectory and the additional parameter. Then we present the scores for each group when the baseline group is added to the ‘higher’ risk group. This analysis was adjusted for sex, paternal depression, maternal education at birth, parity and maternal smoking in pregnancy. Intercepts are centred to age 16, the mean age of all assessments.

ANTD, antenatal depression; PNTD, postnatal depression.

a. linear slope.

b. quadratic slope.

c. cubic change in speed of slope.

d. quartic slope.

### Main results

#### Exposure to maternal ANTD and PNTD

**Fig. 1:**
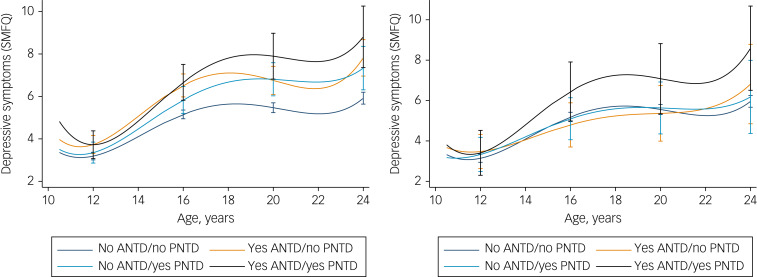
Growth curves to demonstrate the different trajectories of offspring depressive symptom course, determined by the presence of maternal ANTD and/or PNTD exposure (left-hand side) and paternal ANTD and/or PNTD exposure (right-hand side). The 95% CIs are plotted at ages 12, 16, 20 and 24 years.

Using the no ANTD and no PNTD group as a reference (Fig. 1 and Table 2), exposure to both maternal ANTD and PNTD placed offspring in the highest risk group overall. The intercept score for the baseline group was 5.35 (95% CI 5.14–5.62, *P* < 0.01), and the linear rate of change was 0.33 (95% CI 0.29–0.37, *P* < 0.01). Thus, from here on, the β^diff^ value refers to the difference in intercept and slope terms compared with this baseline group, and a higher β^int^ value indicates the score of the comparator group (i.e. the higher risk groups). Having a mother with both ANTD and PNTD resulted in offspring SMFQ scores that were 1.72 points higher at age 16 (β^diff^ = 1.72, 95% CI = 0.84–2.59, *P* < 0.001), producing an intercept value of 7.07 (β^int^ = 7.07, 95% CI = 6.19–7.95, *P* = < 0.001), and the highest linear rate of change in depressive symptom scores per year (*β*^int^ *=* 0.698, 95% CI = 0.47–0.93, *P* = 0.002). In other words, in terms of predicted differences, the results shown in Table 3 indicate that by age 24 the maximum score difference between the two extreme groups was 2.89 points (95% CI = 1.44–4.35, *P* < 0.001). Additional rate of change parameters showed varying effects and are displayed in Table 2, with accompanying predicted differences in Table 3. Exposure to maternal ANTD but not PNTD also resulted in SMFQ scores that were 1.43 points higher at age 16 (β^diff^ = 1.43, 95% CI = 0.87–1.99, *P* < 0.001), producing an intercept value of 6.78 (β^int^ = 6.78, 95% CI = 6.22–7.35, *P* ≤ 0.001); however, there was weak evidence for substantive change over time. In this group, compared with no exposure to either ANTD or PNTD, the difference in scores by age 24 was 1.90 points (1.02–2.78, *P* < 0.001). Finally, exposure to maternal PNTD alone (in the absence of ANTD) resulted in the smallest increase in SMFQ scores, with scores approximately 0.78 points higher at age 16 (β^diff^ = 0.78, 95% CI = 0.11–1.44, *P* = 0.02) and an intercept value of 6.13 (β^int^ = 6.13, 95% CI = 5.47–6.79, *P* = 0.02). However, exposure to PNTD alone was also weakly associated with a higher linear rate of change in depressive symptom scores per year (β^int^ = 0.51, 95% CI = 0.34–0.67, *P* = 0.04).

**Table 3:** Predicted differences in depressive symptoms scores at various ages between maternal antenatal depression (ANTD)/postnatal depression (PNTD) groups and offspring trajectories of depressive symptoms (DS)

Trajectories compared		Predicted mean difference (95% CI) in DS at various ages			
		12 years	16 years	20 years	24 years
No ANTD/no PNTD	Yes ANTD/no PNTD	0.54 (0.14, 0.95), *P* = 0.009	1.37 (0.83, 1.91), *P* < 0.001	1.28 (0.62, 1.95), *P* < 0.001	1.90 (1.02, 2.78), *P* < 0.001
	No ANTD/yes PNTD	0.16 (−0.32, 0.65), *P* = 0.509	0.68 (0.05, 1.31), *P* = 0.035	1.34 (0.55, 2.12), *P* = 0.001	1.42 (0.37, 2.46), *P* = 0.008
	Yes ANTD/yes PNTD	0.54 (−0.10, 1.18), *P* = 0.101	1.51 (0.67, 2.35), *P* < 0.001	2.43 (1.35, 3.51), *P* < 0.001	2.89 (1.44, 4.35), *P* < 0.001
Yes ANTD/no PNTD	No ANTD/yes PNTD	0.38 (−0.23, 0.99), *P* = 0.224	0.69 (−0.12, 1.49), *P* = 0.094	0.05 (−0.94, 1.05), *P* = 0.914	0.49 (−0.84, 1.81), *P* = 0.472
	Yes ANTD/yes PNTD	0.01 (−0.73, 0.75), *P* = 0.987	0.14 (−0.83, 1.12), *P* = 0.770	1.14 (−0.10, 2.39), *P* = 0.072	0.99 (−0.67, 2.66), *P* = 0.243
No ANTD/yes PNTD	Yes ANTD/yes PNTD	0.37 (−0.41, 1.16), *P* = 0.352	0.83 (−0.20, 1.86), *P* = 0.113	1.09 (−0.22, 2.40), *P* = 0.104	1.48 (−0.28, 3.24), *P* = 0.100

Adjusted for sex, paternal depression, maternal education at birth, parity and maternal smoking during pregnancy. The total *N* for this analysis was 5029. The numbers in each group were as follows: no ANTD/no PNTD, 4264; yes ANTD/no PNTD, 365; no ANTD/yes PNTD, 253; yes ANTD/yes PNTD, 147.

#### Exposure to paternal ANTD and PNTD

Using the no ANTD and no PNTD group as a reference (see Fig. 1, where the intercept for this baseline group was 5.40 and the linear rate of change was 0.35, and Table 4), it was clear that overall exposure to both paternal ANTD and PNTD placed offspring in the highest risk group, with SMFQ scores that were 1.37 points higher at age 16 (β^diff^ = 1.37, 95% CI = −0.15–2.89, *P* = 0.08), producing an intercept of 6.77 (β^int^ = 6.77, 95% CI = 5.25–8.296, *P* = 0.08). In terms of predicted differences, the results shown in Table 5 demonstrate that by age 24, the maximum score difference between the two groups was 2.63 (95% CI 0.54–4.72, *P* = 0.014). However, exposure to either paternal ANTD alone or paternal PNTD alone showed little association with higher depressive symptoms. In terms of rate of change of depressive symptoms, there was little evidence of a difference across groups, although the trends were similar to those observed in the maternal data (with rate of change following paternal PNTD alone being greater than that following paternal ANTD alone).

**Table 4:** Paternal adjusted trajectories of offspring depressive symptoms (*N*= 4534)

Parameter	Beta	95% CI		s.e.	*P*-value	Baseline + risk group score parameter	95% CI + risk group score parameter	
No ANTD/no PNTD intercept	5.402	5.171	5.632	0.118	<0.001	–	–	–
No ANTD/no PNTD age^a^	0.349	0.311	0.388	0.019	<0.001	–	–	–
No ANTD/no PNTD age^2^b^	−0.090	−0.101	−0.078	0.006	<0.001	–	–	–
No ANTD/no PNTD age^3^c^	−0.005	−0.006	−0.004	0.001	<0.001	–	–	–
No ANTD/no PNTD age^4^d^	0.002	0.001	0.002	0.0001	<0.001	–	–	–
Yes ANTD/no PNTD intercept	−0.450	−1.586	0.686	0.580	0.438	4.952	3.811	6.093
Yes ANTD/no PNTD age^a^	−0.082	−0.375	0.211	0.150	0.582	0.267	−0.023	0.558
Yes ANTD/no PNTD age^2^b^	0.042	−0.053	0.137	0.048	0.391	−0.048	−0.142	0.046
Yes ANTD/no PNTD age^3^c^	0.003	−0.005	0.010	0.004	0.498	−0.003	−0.010	0.005
Yes ANTD/no PNTD age^4^d^	−0.0005	−0.002	0.001	0.001	0.625	0.001	−0.001	0.003
No ANTD/yes PNTD intercept	−0.121	−1.195	0.952	0.548	0.825	5.280	4.202	6.359
No ANTD/yes PNTD age^a^	−0.044	−0.328	0.239	0.145	0.759	0.305	0.024	0.586
No ANTD/yes PNTD age^2^b^	0.029	−0.059	0.117	0.045	0.524	−0.061	−0.148	0.026
No ANTD/yes PNTD age^3^c^	0.002	−0.005	0.010	0.004	0.528	−0.003	−0.010	0.005
No ANTD/yes PNTD age^4^d^	−0.001	−0.002	0.001	0.001	0.505	0.001	−0.001	0.003
Yes ANTD/yes PNTD intercept	1.370	−0.150	2.890	0.775	0.077	6.772	5.247	8.296
Yes ANTD/yes PNTD age^a^	0.180	−0.198	0.558	0.193	0.351	0.529	0.153	0.906
Yes ANTD/yes PNTD age^2^b^	−0.045	−0.167	0.078	0.063	0.476	−0.134	−0.256	−0.012
Yes ANTD/yes PNTD age^3^c^	−0.002	−0.012	0.008	0.005	0.691	−0.007	−0.017	0.003
Yes ANTD/yes PNTD age^4^d^	0.001	−0.001	0.003	0.001	0.397	0.003	0.0001	0.005

The No ANTD/no PNTD variable is the baseline (reference) group. There are four groups. Each group has an intercept and four terms resulting in four trajectories per group. The different terms (a–d) for each group are further parameters to account for the non-linearity of the trajectories as seen by the fact that the slope is steeper at different ages. For further information on the nature of change in rate of depressive symptoms as per the four terms at different ages, please see the Supplementary material. The intercept for each group was determined by manually adding the baseline intercept for No ANTD/no PNTD to the intercept of the group being compared. Similarly, the rate of change for each subsequent group was determined by adding the rate of change for the baseline group to the rate of change of the group being compared. This is highlighted in the column entitled ‘baseline + risk group score parameter’. For ease of interpretation, we present the original regression coefficients and their 95% CI, s.e. and *P*-values first. The s.e. and *P*-value represent the difference between the baseline trajectory and the additional parameter. Then we present the scores for each group when the baseline group is added to the ‘higher’ risk group. This analysis was adjusted for sex, maternal depression, paternal education at birth, parity and paternal smoking in pregnancy. Intercepts are centred to age 16, the mean age of all assessments.

ANTD, antenatal depression; PNTD, postnatal depression.

a. linear slope.

b. quadratic slope.

c. cubic change in speed of slope.

d. quartic slope.

**Table 5:** Predicted differences in depressive symptoms scores at various ages between paternal antenatal depression (ANTD)/postnatal depression (PNTD) groups and offspring trajectories of depressive symptoms (DS)

Trajectories compared		Predicted mean difference (95% CI) in DS at various ages			
		12	16	20	24
No ANTD/no PNTD	Yes ANTD/no PNTD	0.33 (−0.50, 1.17), *P* = 0.437	0.39 (−0.69, 1.48), *P* = 0.477	0.20 (−1.18, 1.57), *P* = 0.781	0.85 (−1.11, 2.82), *P* = 0.395
	No ANTD/yes PNTD	0.19 (−0.65, 1.02), *P* = 0.658	0.09 (−0.94, 1.12), *P* = 0.864	0.08 (−1.20, 1.36), *P* = 0.904	0.22 (−1.59, 2.03), *P* = 0.811
	Yes ANTD/yes PNTD	0.27 (−0.84, 1.37), *P* = 0.636	1.26 (−0.19, 2.72), *P* = 0.090	1.52 (−0.22, 3.27), *P* = 0.086	2.63 (0.54, 4.72), *P* = 0.014
Yes ANTD/no PNTD	No ANTD/yes PNTD	0.14 (−1.03, 1.31), *P* = 0.809	0.30 (−1.79, 1.18), *P* = 0.687	0.27 (−2.14, 1.59), *P* = 0.774	0.63 (−2.02, 3.29), *P* = 0.640
	Yes ANTD/yes PNTD	0.07 (−1.30, 1.44), *P* = 0.987	1.66 (−0.15, 3.46), *P* = 0.072	1.72 (−0.48, 3.92), *P* = 0.126	1.78 (−1.07, 4.63), *P* = 0.222
No ANTD/yes PNTD	Yes ANTD/yes PNTD	0.08 (−1.29, 1.45), *P* = 0.911	1.35 (−0.42, 3.12), *P* = 0.135	1.45 (−0.70, 3.59), *P* = 0.187	2.41 (−0.34, 5.16), *P* = 0.085

Adjusted for sex, maternal depression, paternal education at birth, parity and paternal smoking during pregnancy. The total *N* for this analysis was 4534. The numbers in each group were as follows: no ANTD/no PNTD, 4317; yes ANTD/no PNTD, 80; no ANTD/yes PNTD, 87; yes ANTD/yes PNTD, 50.

#### Sex effects

Overall, females had higher SMFQ scores at age 16 and faster rates of change in depressive symptom scores per year compared with male offspring (see Supplementary Figs 2 and 3 and Supplementary Tables 7 and 8 for further detail).

##### Exposure to maternal ANTD and PNTD by sex

Exposure to both maternal ANTD and PNTD placed female offspring in the highest risk group, resulting in SMFQ scores that were 3.997 points higher at age 16 (β^int^ = 8.48, 95% CI = 7.35–9.62, *P* ≤ 0.001) than those for male offspring not exposed at either time (Supplementary Table 7). Female offspring exposed to maternal ANTD only were also at increased risk (β^int^ = 7.92, 95% CI = 7.22–8.63, *P* < 0.001). Male offspring exposed at both times scored 1.73 points higher at age 16 (β^int^ = 6.21, 95% CI = 4.90–7.52, *P* = 0.01) than males with no exposure at either time. There was also some evidence suggesting that male offspring exposed to maternal ANTD only (*β^int^* = 6.13, 95% CI = 5.28–6.98, *P* < 0.001) were also at risk. Linear rate of change in depressive symptoms scores was greater for females exposed to both maternal ANTD and PNTD (*β^int^* = 0.61, 95% CI = 0.30–0.91, *P* = 0.04) compared with males of non-depressed parents. Female offspring exposed to maternal PNTD only demonstrated an increase in rate of change of 0.58 SMFQ points (*β^int^* = 0.58, 95% CI = 0.36–0.795, *P* = 0.008) at each time point. By contrast, a weaker association was seen for rate of change of depressive symptoms for female offspring exposed to maternal ANTD only. For males, a similar trend was observed, with those exposed to both maternal ANTD and PNTD demonstrating greater linear rate of change (*β^int^* = 0.75, 95% CI = 0.41–1.09, *P* = 0.007) than any other male group.

##### Exposure to paternal ANTD and PNTD by sex

In relation to fathers (Supplementary Table 8), exposure to both paternal ANTD and PNTD placed female offspring in the highest risk group, with greater depressive symptoms at age 16 (β^int^ = 9.02, 95% CI = 6.99–11.05, *P* ≤ 0.001). This was followed by exposure to paternal ANTD alone, which produced an increase in depressive symptom scores at age 16 among female offspring (β^int^ = 7.65, 95% CI = 5.93–9.37, *P* ≤ 0.01). Exposure to paternal PNTD alone was also associated with increased depressive symptom scores in female offspring but not to the same degree (β^int^ = 6.62, 95% CI = 5.17–8.07, *P* = 0.005). Associations regarding the rate of change in depressive symptom scores in females were weak in comparison with the maternal estimates. With regards to male offspring, exposure to different timings of paternal depression followed a similar trend as those observed for females, yet associations were much weaker in relation to depressive symptoms at age 16 or rate of change in symptoms.

## Discussion

There is a growing body of evidence linking different time points of parental depression to different child mental health outcomes.^7,9^ However, these have typically been in relation to a specific offspring time point. To the best of our knowledge, this study is the first to explore the potential associations of different timings of maternal and paternal depression with offspring depressive symptoms over an extended period of childhood, adolescence and young adulthood using growth-curve analyses. We explored the overall level of offspring depression in terms of an intercept point and the rate of change in depressive symptoms (as demonstrated by the slope of the growth curve). We explored patterns of data in the context of several proposed (and not mutually exclusive) mechanisms; specifically, that ANTD in the mother may be linked genetically but also via possible fetal programming pathways facilitated by the placenta. By contrast, although ANTD in fathers may contribute equally to a genetic effect, it would not share mechanisms via the placenta.^1,7,9^ We also explored the nature of the associations with PNTD, which may relate more to ongoing rates of depression in offspring due to continued exposure of parental symptoms over the course of development.

### Strengths and limitations

Strengths of this study include the comparison of two perinatal depression exposure time points (ANTD and PNTD) in mothers and fathers, thus building on previous research which has been limited by only single time points of parental perinatal depression being available. This allowed us to explore several potential pathways in the intergenerational transmission of depression. Further strengths include the longitudinal analysis over a 14 year period of childhood, adolescence and early adulthood, and the self-reported measure of offspring depressive symptoms. To the best of our knowledge, this is also the first study to explore offspring depression trajectories in relation to timings of parental perinatal depression in this way. However, limitations include the low numbers of fathers in the sample, which limited the interpretation of the associated paternal results. In addition, although we used FIML to adjust for missing data, this may represent a source of bias if the data were not missing at random. Moreover, given the observational nature of this study's design, it was not possible to infer causality or explicitly demonstrate the interpretations made.

### Scientific implications and mechanisms

The literature exploring the potential mechanisms linking parental perinatal depressive symptoms to child outcomes considers several possibilities crossing both the antenatal and postnatal time periods. Genetics, prenatal *in utero* exposure, disruption to parent–child attachment, emotion regulation, modelling, family dysfunction and parenting^32^ have all been suggested. Of these possibilities, some are potentially specific to mothers and the antenatal period, including *in utero* or fetal programming effects often attributed to maternal biological changes affecting offspring brain development.^33^ However, definitive conclusions in relation to the fetal programming of offspring depression remains unknown, and further empirical evidence is required.^34,35^ Beyond the antenatal period, additional exposures may occur postnatally (i.e. from parenting, modelling, family dysfunction) and together with genetic effects may result from either parent.

In this study, we found that the accumulation of exposure to both timings of ANTD and PNTD, from mothers and fathers, carried the greatest risk for offspring. This finding is particularly relevant as it suggests that it is the accumulation of different exposure timings (as opposed to one exposure timing over another) that has the greatest influence on offspring depression course later in life. This increased risk, as seen for the maternal ANTD- and PNTD-exposed offspring group, may reflect the potential influence of combined antenatal mechanisms and subsequent environmental exposure with later maternal depression (i.e. transmission through multiple pathways). It is particularly noteworthy that following maternal exposure, we found the maximum difference in offspring depressive symptom scores between the two extreme groups to be 2.89 points at age 24 years. This corresponds to a 0.5 standard deviation and 11% increase in depressive symptoms, which is a larger difference than that which is found in recovery following depression treatment and thus could be considered clinically relevant.^34,35^ These findings add to the existing literature (including studies that have undertaken trajectory analyses) which has historically focused on only one specific time point.^36–40^

In relation to isolated maternal ANTD, there was some evidence for the specificity of effects, with exposed offspring having higher depressive symptom scores at age 16 compared with paternal ANTD or PNTD in either parent. This may be suggestive of further and specific maternal pathways heightening the risk following exposure to maternal ANTD. One such possibility is a fetal programming mechanism which may confer additional risk on top of genetic and environmental or social mechanisms. Some evidence for such a unique ANTD mechanism was also seen when exploring outcome by gender. It was clear that female offspring appeared to be at greatest risk overall, with higher depressive symptom scores than males across a variety of exposures, but especially so in relation to ANTD exposure. Although there were clear associations between maternal and paternal ANTD and female depression, it is possible that the underlying mechanisms involved are different. For example, there is accumulating evidence that female offspring are more vulnerable to prenatal maternal ‘stress’, and that this effect may be mediated by glucocorticoid mechanisms.^41,42^ However, given the lack of direct physiological connection to the fetus *in utero*, paternal ANTD associated risk is more likely to reflect a shared genetic liability. However, we also found some evidence that male offspring exposed to maternal ANTD (but not maternal PNTD) were at increased risk. These findings differ from previous research, which has found maternal ANTD to be associated with depression in females but not males.^43–45^ These findings, if replicated, may suggest an aspect of male offspring risk that has not been previously appreciated.

We also found evidence suggesting that exposure to maternal PNTD alone (as opposed to maternal ANTD alone) was associated with a greater rate of change in depressive symptom scores in offspring. This effect was directly observed when comparing the ANTD-only maternal analysis with the PNTD-only maternal analysis. In addition, we noted that although exposure to maternal ANTD alone was associated with greater depressive symptoms at age 16 (but not a heightened rate of change), the effect on the linear rate of change in depressive symptoms was not seen until inclusion of maternal PNTD (presence of both maternal ANTD and PNTD), which further suggests a role of maternal PNTD in relation to the linear rate of change in depressive symptoms. This effect was not seen in those offspring exposed to paternal depression, although as previously mentioned this may have been due to the low power of the analysis. From a pragmatic perspective, however, these findings could be explained by the potential effects of postnatal depression on parental emotions, cognitions and behaviour. These in turn have been linked to parenting, which is considered to be one of the most important mediators on the pathway leading to intergenerational transmission of mental health.^1^

### Clinical implications

The results suggested in this paper highlight that young people exposed to both parental ANTD and PNTD may represent a vulnerable group. We also observed a dynamic effect of maternal PNTD on the rate of change of depressive symptoms in offspring, potentially highlighting a role for the development and delivery of interventions seeking to buffer the effects of parental mental illness during childhood and adolescence.

### Interpretation

This study found that offspring exposed to both parental ANTD and PNTD were at greatest risk of depressive symptoms. We also found some evidence that could be consistent with the idea that although different timings of parental perinatal depression share some risk mechanisms, there may be additional and different ANTD pathways involved. Finally, we observed a specific effect of maternal PNTD exposure on the rate of change of offspring depressive symptoms over time. However, these interpretations need to be directly tested, and replication with larger numbers is required, in particular, for the paternal analyses.

## Data Availability

P.R., A.S.F.K. and R.M.P. had access to the study data, and access is currently ongoing. The data were obtained from ALSPAC. Access to these data can be requested through application to the ALSPAC executive via the online proposal system (http://www.bristol.ac.uk/alspac/researchers/access/).

## Supplementary material

For supplementary material accompanying this paper visit http://dx.doi.org/10.1192/bjo.2021.959. click here to view supplementary material
