# Supplementary material for: Maternal and paternal depression and child mental health trajectories: evidence from the Avon Longitudinal Study of Parents and Children
Source: BJPsych Open. 2021 Sep 24;7(5):e166. doi: 10.1192/bjo.2021.959 (PMC8485341; doi:10.1192/bjo.2021.959)
Supplement: Supplementary file 1 [file S2056472421009595sup001.docx]

**Supplementary Materials**

Please note that the supplementary figures and tables below have been numbered in line with their order of appearance in the main manuscript text.

**Statistical Methods**

Let $y_{ij}$ denote the depressive symptom score for individual $j$ at occasion $i$. The simplest trajectory model consists of a random intercept and a random quartic polynomial comprised of four age terms: age, age^2^, age^3^ and age^4^. The model can then be written as:

$$y_{ij}=\beta_{0}+\beta_{1}t_{ij}+\beta_{2}t_{ij}^{2}+\beta_{3}t_{ij}^{3}+\beta_{4}t_{ij}^{4}+u_{0j}+u_{1j}t_{ij}+u_{2j}t_{ij}^{2}+u_{3j}t_{ij}^{3}+u_{4j}t_{ij}^{4}+e_{ij}$$

*(Supplementary Equation 1)*

where $t_{ij}$ denotes the age in years (centred around 16.53 years, the mean age of all assessments^[[1]](#footnote-1)^) for that individual at that occasion, $t_{ij}^{2}$, $t_{ij}^{3}$, and $t_{ij}^{4}$, denote the quadratic, cubic and quartic age terms, and where $\beta_{0},\beta_{1},\beta_{2},\beta_{3},\beta_{4}$ denote the associated regression coefficients. The $u_{0j}$, $u_{1j}$, $u_{2j}$, $u_{3j}$ and $u_{4j}$ are the random intercept, linear, quadratic, cubic and quartic individual-specific effects, and $e_{ij}$ is the occasion-specific residual.

The random effects are assumed multivariate normal distributed with zero mean vector and constant covariance matrix:

$$\left( \begin{aligned} u_{0j} \\ u_{1j} \\ u_{2j} \\ u_{3j} \\ u_{4j} \end{aligned} \right)\sim N\left\{ \left( \begin{aligned} 0 \\ 0 \\ 0 \\ 0 \\ 0 \end{aligned} \right), \left( \begin{aligned} \sigma_{u0}^{2} \\ \sigma_{u01} \\ \sigma_{u02} \\ \sigma_{u03} \\ \sigma_{u04} \end{aligned}\begin{aligned} \\ \sigma_{u1}^{2} \\ \sigma_{u12} \\ \sigma_{u13} \\ \sigma_{u14} \end{aligned}\begin{aligned} \\ \\ \sigma_{u2}^{2} \\ \sigma_{u23} \\ \sigma_{u24} \end{aligned}\begin{aligned} \\ \\ \\ \sigma_{u3}^{2} \\ \sigma_{u34} \end{aligned}\begin{aligned} \\ \\ \\ \\ \sigma_{u4}^{2} \end{aligned} \right) \right\}$$

*(Supplementary Equation 2)*

The elements of the covariance matrix summarise the degree to which individual-specific trajectories vary around the population-averaged trajectory. The residuals are assumed normally distributed with zero mean and constant variance:

$$e_{ij}\sim N(0, \sigma_{e}^{2})$$

*(Supplementary Equation 3)*

**Predicted Depressive Symptom Trajectories**

The model is therefore set up to predict both the population average trajectory

$$E\left( y_{ij} | t_{ij} \right)=\beta_{0}+\beta_{1}t_{ij}+\beta_{2}t_{ij}^{2}+\beta_{3}t_{ij}^{3}+\beta_{4}t_{ij}^{4}$$

*(Supplementary Equation 4)*

and individual specific trajectories

$$E\left( y_{ij} | t_{ij},u_{0j},u_{1j},u_{2j},u_{3j},u_{4j} \right)=\beta_{0}+\beta_{1}t_{ij}+\beta_{2}t_{ij}^{2}+\beta_{3}t_{ij}^{3}+\beta_{4}t_{ij}^{4}+u_{0j}+u_{1j}t_{ij}+u_{2j}t_{ij}^{2}+u_{3j}t_{ij}^{3}+u_{4j}t_{ij}^{4}$$

*(Supplementary Equation 5)*

Substituting the parameter estimates and the predicted random effect values into the above expression gives the population average and individual specific predicted depressive symptom scores.

**Association Between Parental Depression and Offspring Trajectories of Depressive Symptoms**

We ran a model to examine the association between parental depression (No ANTD/No PNTD, Yes ANTD/No PNTD, No ANTD/Yes PNTD and Yes ANTD/Yes PNTD) and offspring trajectories of depressive symptoms. We conduced separate analysis for maternal and paternal depression.

We decided to treat parental depression as a four-category ordinal variable, rather than as a linear variable as the difference in depressive symptoms between parental depression may not increase linearly and it is contested whether ANTD or PNTD is more detrimental to offspring mental health.

First, we created three dummy variables corresponding to the level of parental depression. The dummy variables were then entered as main effects and as interactions with the four age terms of the quartic polynomial (i.e., parental depression level X intercept; parental depression level X age; parental depression level X age^2^; parental depression level X age^3^ and parental depression level X age^4^). The intercept and the main effects of the quartic age polynomial therefore describe the No ANTD/No PNTD group (i.e., the baseline or reference trajectory). Then, for any given parental depression level, the corresponding dummy variable and interactions with the four age terms of the quartic age polynomial describe how the trajectory for that parental depression group deviates from that of the bassline no parental depression group.

Let $x_{j}$ denote the level of parental depression (0 [No ANTD/No PNTD], 1 [Yes ANTD/No PNTD], 2 [No ANTD/Yes PNTD], 3 [Yes ANTD/Yes PNTD]) and $x_{0j},x_{1j},x_{2j},x_{3j}$ the series of three dummy variables derived from this. The following table shows the relationship between the dummy variables and the original ordinal variable.

| $x_{j}$ | $x_{0j}$ | $x_{1j}$ | $x_{2j}$ | $x_{3j}$ |
| --- | --- | --- | --- | --- |
| 0 | 1 | 0 | 0 | 0 |
| 1 | 0 | 1 | 0 | 0 |
| 2 | 0 | 0 | 1 | 0 |
| 3 | 0 | 0 | 0 | 1 |

$x_{0j}$ represents the baseline trajectory for the model for No ANTD/No PNTD. When $x_{1j}$ is set to 1 ($x_{2j}$ and $x_{3j}$ are set to 0), the model gives the trajectory with Yes ANTD/No PNTD. When $x_{2j}$ is set to 1 ($x_{1j}$ and $x_{3j}$ are set to 0), the model gives the No ANTD/Yes PNTD trajectory, and when $x_{3j}$ is set to 1 ($x_{1j}$ and $x_{2j}$ are set to 0), the model gives the Yes ANTD/Yes PNTD trajectory.

The depressive symptom score for individual $j$ at occasion $i$ is therefore given by:

$$y_{ij}=\beta_{0}+\beta_{1}t_{ij}+\beta_{2}t_{ij}^{2}+\beta_{3}t_{ij}^{3}+\beta_{4}t_{ij}^{4}$$

$$+ \beta_{5}x_{1j}+\beta_{6}x_{1j}t_{ij}+\beta_{7}x_{1j}t_{ij}^{2}+\beta_{8}x_{1j}t_{ij}^{3} +\beta_{9}x_{1j}t_{ij}^{4}$$

$$+ \beta_{10}x_{2j}+ \beta_{11}x_{2j}t_{ij}+\beta_{12}x_{2j}t_{ij}^{2}+\beta_{13}x_{2j}t_{ij}^{3} +\beta_{14}x_{2j}t_{ij}^{4}$$

$$+ \beta_{15}x_{3j}+ \beta_{16}x_{3j}t_{ij}+\beta_{17}x_{3j}t_{ij}^{2}+\beta_{18}x_{3j}t_{ij}^{3} +\beta_{19}x_{3j}t_{ij}^{4}$$

$$+u_{0j}+u_{1j}t_{ij}+u_{2j}t_{ij}^{2}+u_{3j}t_{ij}^{3} +u_{4j}t_{ij}^{4}+e_{ij}$$

*(Supplementary Equation 6)*

The population average depressive symptom score for each parental depression group can then be obtained by setting all random effects to 0 and by setting the dummy variables to their relevant values as illustrated in the table above. An alternative way to visualise the population-average for each trajectory can be written as this:

No ANTD/No PNTD: $E\left( y_{ij} | t_{ij} \right)= \beta_{0}+\beta_{1}t_{ij}+\beta_{2}t_{ij}^{2}+\beta_{3}t_{ij}^{3}+\beta_{4}t_{ij}^{4}$
Yes ANTD/No PNTD: $E\left( y_{ij} | t_{ij}, x_{0j}=0, x_{1j}=1, x_{2j}:x_{3j}=0 \right)= {(\beta}_{0}+\beta_{5}) +{(\beta}_{1}+ \beta_{6})t_{ij}+{(\beta}_{2}+ \beta_{7})t_{ij}^{2}+{(\beta}_{3}+ \beta_{8})t_{ij}^{3}+{(\beta}_{4}+ \beta_{9})t_{ij}^{4}$
No ANTD/Yes PNTD: $E\left( y_{ij} | t_{ij}, x_{0j}: x_{1j}=0, x_{2j}=1, x_{3j}=0 \right)= {(\beta}_{0}+\beta_{10}) +{(\beta}_{1}+ \beta_{11})t_{ij}+{(\beta}_{2}+ \beta_{12})t_{ij}^{2}+{(\beta}_{3}+ \beta_{13})t_{ij}^{3}+{(\beta}_{4}+ \beta_{14})t_{ij}^{4}$
Yes ANTD/Yes PNTD: $E\left( y_{ij} | t_{ij}, x_{0j}:x_{2j}=0, x_{3j}=1 \right)= {(\beta}_{0}+\beta_{15}) +{(\beta}_{1}+ \beta_{16})t_{ij}+{(\beta}_{2}+ \beta_{17})t_{ij}^{2}+{(\beta}_{3}+ \beta_{18})t_{ij}^{3}+{(\beta}_{4}+ \beta_{19})t_{ij}^{4}$

*(Supplementary Equation 7)*

**Association Between Parental Depression and Offspring Trajectories of Depressive Symptoms by Sex**

We ran a model to examine the association between parental depression (No ANTD/No PNTD, Yes ANTD/No PNTD, No ANTD/Yes PNTD and Yes ANTD/Yes PNTD) and offspring trajectories of depressive symptoms by sex. Again, we conduced separate analysis for maternal and paternal depression.

As in the previous analysis, we treated parental depression as a four-category ordinal variable (i.e., No ANTD/No PNTD, Yes ANTD/No PNTD, No ANTD/Yes PNTD and Yes ANTD/Yes PNTD). However, in this analysis we created dummy variables corresponding to the level of parental depression that were split by sex (i.e., i.e., male No ANTD/No PNTD, male Yes ANTD/No PNTD, male No ANTD/Yes PNTD, male Yes ANTD/Yes PNTD, female No ANTD/No PNTD, female Yes ANTD/No PNTD, female No ANTD/Yes PNTD and female Yes ANTD/Yes PNTD. These dummy variables were then entered as main effects and as interactions with the four age terms of the quartic polynomial (i.e., sex/parental depression level X intercept; parental depression level X age; parental depression level X age^2^; parental depression level X age^3^ and parental depression level X age^4^). The intercept and the main effects of the quartic age polynomial therefore describe the male No ANTD/No PNTD group (i.e., the baseline or reference trajectory). Then, for any given sex/parental depression level, the corresponding dummy variable and interactions with the four age terms of the quartic age polynomial describe how the trajectory for that parental depression group deviates from that of the baseline male no parental depression group.

Let $x_{j}$ denote the level of parental depression with (0 [male No ANTD/No PNTD], 1 [male Yes ANTD/No PNTD], 2 [male No ANTD/Yes PNTD], 3 [male Yes ANTD/Yes PNTD], 4 [female No ANTD/No PNTD], 5 [female Yes ANTD/No PNTD], 6 [female No ANTD/Yes PNTD], 7 [female Yes ANTD/Yes PNTD]) and ${x_{0j},x}_{1j},x_{2j},x_{3j}, x_{4j},x_{5j},x_{6j}, x_{7j}$ the series of dummy variables derived from this. The following table shows the relationship between the dummy variables and the original ordinal variable.

| $x_{j}$ | $x_{0j}$ | $x_{1j}$ | $x_{2j}$ | $x_{3j}$ | $x_{4j}$ | $x_{5j}$ | $x_{6j}$ | $x_{7j}$ |
| --- | --- | --- | --- | --- | --- | --- | --- | --- |
| 0 | 1 | 0 | 0 | 0 | 0 | 0 | 0 | 0 |
| 1 | 0 | 1 | 0 | 0 | 0 | 0 | 0 | 0 |
| 2 | 0 | 0 | 1 | 0 | 0 | 0 | 0 | 0 |
| 3 | 0 | 0 | 0 | 1 | 0 | 0 | 0 | 0 |
| 4 | 0 | 0 | 0 | 0 | 1 | 0 | 0 | 0 |
| 5 | 0 | 0 | 0 | 0 | 0 | 1 | 0 | 0 |
| 6 | 0 | 0 | 0 | 0 | 0 | 0 | 1 | 0 |
| 7 | 0 | 0 | 0 | 0 | 0 | 0 | 0 | 1 |

$x_{0j}$ represents the baseline trajectory for the model for male No ANTTD/No PNTD. When $x_{1j}$ is set to 1 the model gives the trajectory with male Yes AND/No PNTD. When $x_{2j}$ is set to 1 the model gives the male No ANTD/Yes PNTD trajectory. When $x_{3j}$ is set to 1 the model gives the male Yes ANTD/Yes PNTD trajectory. When $x_{4j}$ is set to 1 the model gives the trajectory with female No ANTD/No PNTD. When $x_{5j}$ is set to 1 the model gives the female Yes ANTD/No PNTD trajectory. When $x_{6j}$ is set to 1 the model gives the female No ANTD/Yes PNTD trajectory. When $x_{7j}$ is set to 1 the model gives the female Yes ANTD/Yes PNTD trajectory.

The depressive symptom score for individual $j$ at occasion $i$ is therefore given by:

$$y_{ij}=\beta_{0}+\beta_{1}t_{ij}+\beta_{2}t_{ij}^{2}+\beta_{3}t_{ij}^{3}+\beta_{4}t_{ij}^{4}$$

$$+ \beta_{5}x_{1j}+\beta_{6}x_{1j}t_{ij}+\beta_{7}x_{1j}t_{ij}^{2}+\beta_{8}x_{1j}t_{ij}^{3} +\beta_{9}x_{1j}t_{ij}^{4}$$

$$+ \beta_{10}x_{2j}+ \beta_{11}x_{2j}t_{ij}+\beta_{12}x_{2j}t_{ij}^{2}+\beta_{13}x_{2j}t_{ij}^{3} +\beta_{14}x_{2j}t_{ij}^{4}$$

$$+ \beta_{15}x_{3j}+ \beta_{16}x_{3j}t_{ij}+\beta_{17}x_{3j}t_{ij}^{2}+\beta_{18}x_{3j}t_{ij}^{3} +\beta_{19}x_{3j}t_{ij}^{4}$$

$$+ \beta_{20}x_{4j}+\beta_{21}x_{4j}t_{ij}+\beta_{22}x_{4j}t_{ij}^{2}+\beta_{23}x_{4j}t_{ij}^{3} +\beta_{24}x_{4j}t_{ij}^{4}$$

$$+ \beta_{25}x_{5j}+ \beta_{26}x_{5j}t_{ij}+\beta_{27}x_{5j}t_{ij}^{2}+\beta_{28}x_{5j}t_{ij}^{3} +\beta_{29}x_{5j}t_{ij}^{4}$$

$$+ \beta_{30}x_{6j}+ \beta_{31}x_{6j}t_{ij}+\beta_{32}x_{6j}t_{ij}^{2}+\beta_{33}x_{6j}t_{ij}^{3} +\beta_{34}x_{6j}t_{ij}^{4}$$

$$+ \beta_{35}x_{7j}+ \beta_{36}x_{7j}t_{ij}+\beta_{37}x_{7j}t_{ij}^{2}+\beta_{38}x_{7j}t_{ij}^{3} +\beta_{39}x_{7j}t_{ij}^{4}$$

$$+u_{0j}+u_{1j}t_{ij}+u_{2j}t_{ij}^{2}+u_{3j}t_{ij}^{3} +u_{4j}t_{ij}^{4}+e_{ij}$$

*(Supplementary Equation 8)*

The population average depressive symptom score for each parental depression group can then be obtained by setting all random effects to 0 and by setting the dummy variables to their relevant values as illustrated in the table above. An alternative way to visualise the population-average for each trajectory can be written as this:

Male No A/No P: $E\left( y_{ij} | t_{ij} \right)= \beta_{0}+\beta_{1}t_{ij}+\beta_{2}t_{ij}^{2}+\beta_{3}t_{ij}^{3}+\beta_{4}t_{ij}^{4}$
Male Yes A/No P: $E\left( y_{ij} | t_{ij}, x_{0j}=0, x_{1j}=1, x_{2j}:x_{7j}=0 \right)= {(\beta}_{0}+\beta_{5}) +{(\beta}_{1}+ \beta_{6})t_{ij}+{(\beta}_{2}+ \beta_{7})t_{ij}^{2}+{(\beta}_{3}+ \beta_{8})t_{ij}^{3}+{(\beta}_{4}+ \beta_{9})t_{ij}^{4}$
Male No A/Yes P: $E\left( y_{ij} | t_{ij}, x_{0j}:x_{1j}=0, x_{2j}=1, x_{3j}:x_{7j}=0 \right)= {(\beta}_{0}+\beta_{10}) +{(\beta}_{1}+ \beta_{11})t_{ij}+{(\beta}_{2}+ \beta_{12})t_{ij}^{2}+{(\beta}_{3}+ \beta_{13})t_{ij}^{3}+{(\beta}_{4}+ \beta_{14})t_{ij}^{4}$
Male Yes A/Yes P: $E\left( y_{ij} | t_{ij}, x_{0j}:x_{2j}=0, x_{3j}=1, x_{4j}:x_{7j}=0 \right)= {(\beta}_{0}+\beta_{15}) +{(\beta}_{1}+ \beta_{16})t_{ij}+{(\beta}_{2}+ \beta_{17})t_{ij}^{2}+{(\beta}_{3}+ \beta_{18})t_{ij}^{3}+{(\beta}_{4}+ \beta_{19})t_{ij}^{4}$
Female No A/No P: $E\left( y_{ij} | t_{ij}, x_{0j}: x_{3j}=0, x_{4j}=1, x_{5j}:x_{7j}=0 \right)= {(\beta}_{0}+\beta_{20}) +{(\beta}_{1}+ \beta_{21})t_{ij}+{(\beta}_{2}+ \beta_{22})t_{ij}^{2}+{(\beta}_{3}+ \beta_{23})t_{ij}^{3}+{(\beta}_{4}+ \beta_{24})t_{ij}^{4}$
Female Yes A/No P: $E\left( y_{ij} | t_{ij}, x_{0j}: x_{4j}=0, x_{5j}=1, x_{6j}:x_{7j}=0 \right)= {(\beta}_{0}+\beta_{25}) +{(\beta}_{1}+ \beta_{26})t_{ij}+{(\beta}_{2}+ \beta_{27})t_{ij}^{2}+{(\beta}_{3}+ \beta_{28})t_{ij}^{3}+{(\beta}_{4}+ \beta_{29})t_{ij}^{4}$
Female No A/Yes P: $E\left( y_{ij} | t_{ij}, x_{0j}: x_{5j}=0, x_{6j}=1, x_{7j}=0 \right)= {(\beta}_{0}+\beta_{30}) +{(\beta}_{1}+ \beta_{31})t_{ij}+{(\beta}_{2}+ \beta_{32})t_{ij}^{2}+{(\beta}_{3}+ \beta_{33})t_{ij}^{3}+{(\beta}_{4}+ \beta_{34})t_{ij}^{4}$
Female Yes A/Yes P: $E\left( y_{ij} | t_{ij}, x_{0j}: x_{6j}=0, x_{7j}=1 \right)= {(\beta}_{0}+\beta_{35}) +{(\beta}_{1}+ \beta_{36})t_{ij}+{(\beta}_{2}+ \beta_{37})t_{ij}^{2}+{(\beta}_{3}+ \beta_{38})t_{ij}^{3}+{(\beta}_{4}+ \beta_{39})t_{ij}^{4}$

*(Supplementary Equation 9)*

**Model Fit**

Model fit for the trajectories were assessed using likelihood ratio tests and model fit statistics.

We started by building a stepwise model which tested which model was preferred. In order to achieve this, we first created trajectories with only one linear polynomial term: “Age” (Figure S4.A). We then added a quadratic polynomial term into the model: “Age^2^” (Figure S4.B). A likelihood ratio test suggested that this second model was preferred to the linear model ($x$^2^ = 1383.27, p <.001). We then added a cubic polynomial term to the model: “Age^3^” (Figure S4.C), and a likelihood ratio test suggested this model was preferred to the quadratic model ($x$^2^ = 394.1, p <.001). We then added a quartic polynomial term to the model: “Age^4^” (Figure S4.D), and a likelihood ratio test suggested this model was preferred to the cubic model ($x$^2^ = 858.25, p <.001).

We also examined model fit using statistics such as: deviance, Akaike information criterion (AIC) and Bayesian information criterion (BIC), as recommended by (Singer & Willett, 2003). Briefly, lower deviance, AIC and BIC indicate better model fit.

We examined model four times: we examined model fit for just those with at least one assessment of the SMFQ (Table S11). Then, we examined whether the shape of the trajectory differed between individuals with at least one SMFQ assessment, compared to at least four SMFQ assessments (Table S2, Figure S1). We also examined model fit for individuals with at least one SMFQ assessment and data on maternal depression and the confounders (Table S12). Finally, we examined model fit for individuals with at least one SMFQ assessment and data on paternal depression and confounders (Table S13). All analyses indicted that a quartic polynomial model fitted the data the best.

| Table S1. Descriptive statistics and reliability of the Short Mood and Feelings Questionnaire (SMFQ). | | | | | | | |
| --- | --- | --- | --- | --- | --- | --- | --- |
| Occasion | **Mean Age** | **Sample Size** | **Mean SMFQ** | **SMFQ SD** | **Above SMFQ Threshold (≥11)** | **α** | **Source of SMFQ** |
| 1 | 10.65 | 7,364 | 4.04 | 3.51 | 5.96% | 0.8 | Clinic |
| 2 | 12.81 | 6,716 | 3.97 | 3.86 | 7.10% | 0.84 | Clinic |
| 3 | 13.84 | 6,019 | 4.92 | 4.49 | 11.66% | 0.87 | Clinic |
| 4 | 16.68 | 4,997 | 5.91 | 5.64 | 18.05% | 0.91 | Questionnaire |
| 5 | 17.84 | 4,497 | 6.59 | 5.25 | 21.64% | 0.9 | Clinic |
| 6 | 18.65 | 3,335 | 6.83 | 5.93 | 21.86% | 0.91 | Questionnaire |
| 7 | 21.95 | 3,305 | 5.7 | 5.58 | 18.06% | 0.92 | Questionnaire |
| 8 | 22.88 | 3,856 | 6.21 | 5.55 | 18.80% | 0.91 | Questionnaire |
| 9 | 23.8 | 3,915 | 7.03 | 6.06 | 24.75% | 0.91 | Questionnaire |

Note: This table reflects the descriptive statistics for each wave of outcome measurement (9 time points in total).

Abbreviations: SMFQ- Short Mood and Feelings Questionnaire; SD- Standard Deviation; α: Coefficient alpha estimate of reliability for the SMFQ at each occasion

| Table S2. Comparisons between trajectories with at least 1 SMFQ measure and trajectories with at least 4 SMFQ measures | | | | | |
| --- | --- | --- | --- | --- | --- |
| Model | **Intercept** | **Age** | **Age^2** | **Age^3** | **Age^4** |
| At least 1 SMFQ  (9399) | 6.278 (6.166, 6.391) P < .001 | 0.365 (0.338, 0.393) P < .001 | -0.098 (-0.107, -0.090) P < .001 | -0.005 (-0.006, -0.005) P < .001 | 0.002 (0.001, 0.002) P <.001 |
| At least 4 SMFQ | 6.170 (6.046, 6.294) P < .001 | 0.348 (0.318, 0.377) P < .001 | -0.095 (-0.104, -0.086) P < .001 | -0.005 (-0.006, -0.004) P <.001 | 0.002 (0.001, 0.002) P < .002 |

Note: Model parameters by number of SMFQ measures included. SMFQ: short mood and feelings questionnaire.

**Figure S1 – Comparison between trajectories with at least 1 measurement of the SMFQ and trajectories with at least 4 measurements of the SMFQ.**

***Abbreviations: SMFQ- short mood and feelings questionnaire; DS-depressive symptoms.**

| Parameter | Beta | 95% CIs | | Std. Err. | P-Value |
| --- | --- | --- | --- | --- | --- |
| No ANTD / No PNTD Intercept | 5.228 | 4.970 | 5.487 | 0.132 | <.001 |
| No ANTD / No PNTD Age ^a^ | 0.305 | 0.258 | 0.352 | 0.024 | <.001 |
| No ANTD / No PNTD Age^2 ^b^ | -0.077 | -0.092 | -0.063 | 0.007 | <.001 |
| No ANTD / No PNTD Age^3 ^c^ | -0.004 | -0.006 | -0.003 | 0.001 | <.001 |
| No ANTD / No PNTD Age^4 ^d^ | 0.001 | 0.001 | 0.002 | 0.0001 | <.001 |
| Yes ANTD / No PNTD Intercept | 1.427 | 0.722 | 2.132 | 0.360 | <.001 |
| Yes ANTD / No PNTD Age ^a^ | 0.132 | -0.047 | 0.311 | 0.091 | 0.148 |
| Yes ANTD / No PNTD Age^2 ^b^ | -0.062 | -0.115 | -0.008 | 0.027 | 0.024 |
| Yes ANTD / No PNTD Age^3 ^c^ | -0.003 | -0.008 | 0.002 | 0.002 | 0.235 |
| Yes ANTD / No PNTD Age^4 ^d^ | 0.001 | 0.000 | 0.002 | 0.001 | 0.019 |
| No ANTD / Yes PNTD Intercept | 0.679 | -0.165 | 1.523 | 0.431 | 0.115 |
| No ANTD / Yes PNTD Age ^a^ | 0.102 | -0.112 | 0.315 | 0.109 | 0.351 |
| No ANTD / Yes PNTD Age^2 ^b^ | 0.018 | -0.046 | 0.081 | 0.032 | 0.58 |
| No ANTD / Yes PNTD Age^3 ^c^ | -0.0001 | -0.006 | 0.006 | 0.003 | 0.984 |
| No ANTD / Yes PNTD Age^4 ^d^ | -0.001 | -0.002 | 0.001 | 0.001 | 0.421 |
| Yes AND / Yes PNTD Intercept | 1.950 | 0.826 | 3.074 | 0.573 | 0.001 |
| Yes AND / Yes PNTD Age ^a^ | 0.309 | 0.019 | 0.599 | 0.148 | 0.037 |
| Yes AND / Yes PNTD Age^2 ^b^ | -0.021 | -0.108 | 0.067 | 0.045 | 0.643 |
| Yes AND / Yes PNTD Age^3 ^c^ | -0.004 | -0.012 | 0.004 | 0.004 | 0.315 |
| Yes AND / Yes PNTD Age^4 ^d^ | 0.001 | -0.001 | 0.003 | 0.001 | 0.424 |

**Table S3. Maternal adjusted trajectories of offspring depressive symptoms, adjusted for later time point of maternal depression on the EPDS at age 22 (n-2,708)**

Note: The No ANTD/No PNTD variable is the baseline (reference) group. There are four groups. Each group has an intercept and four terms resulting in four trajectories per group. The different terms (a-d) for each group are further parameters to account for the non-linearity of the trajectories as seen by the fact that the slope is steeper at different ages. Here we present the original regression coefficients and their 95%Cis, standard errors and p values for each group. Intercepts are centred to age 16, the mean age of all assessments.

Abbreviations: EPDS- Edinburgh Postnatal Depression Rating Scale; ANTD- antenatal depression; PNTD-postnatal depression; ^a^- linear slope, ^b^- quadratic slope, ^c^- cubic change in speed of slope, ^d^-quartic slope

| Table S4. Participant demographics for paternal depression exposure | | |  | |
| --- | --- | --- | --- | --- |
|  | **Sample with complete exposure data (paternal depression)^a^**  **N (%)** | **Sample with complete outcome data (at least one offspring depression)^b^**  **N (%)** | | **Sample with complete exposure, outcome and confounding data^c^**  **N (%)** |
| Sex |  |  | |  |
| Males | 3795 (51.08) | 4495 (47.85) | | 2209 (48.72) |
| Females | 3634 (48.92) | 4899 (52.15) | | 2325 (51.28) |
| Paternal Education |  |  | |  |
| A Level or Higher | 3588 (53.70) | 4011 (52.60) | | 2570 (56.68) |
| GCSE/O Level | 3093 (46.30) | 3615 (47.60) | | 1964 (43.32) |
| Parity |  |  | |  |
| First Born | 3491 (47.76) | 3918 (45.94) | | 2239 (49.38) |
| Second Born | 2501 (34.21) | 3041 (35.66) | | 1586 (34.98) |
| Third Born + | 1318 (18.03) | 1569 (18.40) | | 709 (15.64) |
| Paternal Smoking During Pregnancy |  |  | |  |
| No Prenatal Smoking | 4661 (65.79) | 4414 (67.42) | | 3201 (70.60) |
| Yes Postnatal Smoking | 2424 (34.21) | 2133 (32.58) | | 1838 (29.40) |
| Maternal Depression |  |  | |  |
| No ANTD /No PNTD | 5678 (83.65) | 6240 (83.38) | | 3869 (85.33) |
| Yes ANTD/ No PNTD | 524 (7.72) | 582 (7.78) | | 316 (6.97) |
| No ANTD/ Yes PNTD | 346 (5.10) | 387 (5.17) | | 226 (4.98) |
| Yes ANTD/ Yes PNTD | 240 (3.54) | 275 (3.67) | | 123 (2.71) |

Note. ^a^The number of offspring for whom data on maternal perinatal depression was available. Reading down the exposure sample column the figures also indicate the number of offspring who had data available on each individual variable in addition to paternal depression information. ^b^ The number of offspring for whom outcome data of at least one SMFQ result was available. Reading down the outcome sample column the figures also indicate the number of offspring who had data available on each individual variable in addition to having at least one SMFQ result. ^c^The number of offspring for whom data was available on paternal depression, confounding variables and at least one SMFQ result.

Abbreviations: ANTD-antenatal depression; PNTD-post natal depression; GCSE- General Certificate of Secondary Education; A level- General Certificate of Education Advanced level; n= number of offspring.

| Parameter | Beta | 95% CIs | | Std. Err. | P-Value |
| --- | --- | --- | --- | --- | --- |
| No ANTD / No PNTD Intercept | 5.968 | 5.836 | 6.101 | 0.068 | <.001 |
| No ANTD / No PNTD Age ^a^ | 0.333 | 0.300 | 0.366 | 0.017 | <.001 |
| No ANTD / No PNTD Age^2 ^b^ | -0.094 | -0.104 | -0.084 | 0.005 | <.001 |
| No ANTD / No PNTD Age^3 ^c^ | -0.005 | -0.006 | -0.004 | 0.0004 | <.001 |
| No AND / No PNTD Age^4 ^d^ | 0.002 | 0.002 | 0.002 | 0.0001 | <.001 |
| Yes ANTD / No PNTD Intercept | 1.580 | 1.110 | 2.051 | 0.240 | <.001 |
| Yes ANTD / No PNTD Age ^a^ | 0.048 | -0.069 | 0.166 | 0.060 | 0.42 |
| Yes ANTD / No PNTD Age^2 ^b^ | -0.034 | -0.072 | 0.003 | 0.019 | 0.071 |
| Yes ANTD / No PNTD Age^3 ^c^ | 0.0003 | -0.003 | 0.003 | 0.002 | 0.862 |
| Yes ANTD / No PNTD Age^4 ^d^ | 0.001 | -0.0002 | 0.001 | 0.0004 | 0.155 |
| No ANTD / Yes PNTD Intercept | 1.208 | 0.644 | 1.773 | 0.288 | <.001 |
| No ANTD / Yes PNTD Age ^a^ | 0.192 | 0.051 | 0.334 | 0.072 | 0.008 |
| No ANTD / Yes PNTD Age^2 ^b^ | -0.006 | -0.051 | 0.038 | 0.023 | 0.787 |
| No ANTD / Yes PNTD Age^3 ^c^ | -0.003 | -0.006 | 0.001 | 0.002 | 0.145 |
| No ANTD / Yes PNTD Age^4 ^d^ | 0.0001 | -0.001 | 0.001 | 0.0005 | 0.847 |
| Yes ANTD / Yes PNTD Intercept | 1.654 | 0.966 | 2.342 | 0.351 | <.001 |
| Yes ANTD / Yes PNTD Age ^a^ | 0.299 | 0.120 | 0.478 | 0.091 | 0.001 |
| Yes ANTD / Yes PNTD Age^2 ^b^ | -0.009 | -0.065 | 0.047 | 0.029 | 0.758 |
| Yes ANTD / Yes PNTD Age^3 ^c^ | -0.006 | -0.010 | -0.001 | 0.002 | 0.015 |
| Yes ANTD / Yes PNTD Age^4 ^d^ | 0.001 | -0.0004 | 0.002 | 0.001 | 0.232 |

**Table S5. Maternal unadjusted trajectories of offspring depressive symptoms (n=7,484**)

Note: The No ANTD/No PNTD variable is the baseline (reference) group. There are four groups. Each group has an intercept and four terms resulting in four trajectories per group. The different terms (a-d) for each group are further parameters to account for the non-linearity of the trajectories as seen by the fact that the slope is steeper at different ages. Here we present the original regression coefficients and their 95%Cis, standard errors and p values for each group. Intercepts are centred to age 16, the mean age of all assessments.

Abbreviations: ANTD- antenatal depression; PNTD-postnatal depression; ^a^- linear slope, ^b^- quadratic slope, ^c^- cubic change in speed of slope, ^d^-quartic slope

| Parameter | Beta | 95% CIs | | Std. Err. | P-Value |
| --- | --- | --- | --- | --- | --- |
| No ANTD / No PNTD Intercept | 6.124 | 5.982 | 6.265 | 0.072 | <.001 |
| No ANTD / No PNTD Age ^a^ | 0.360 | 0.325 | 0.395 | 0.018 | <.001 |
| No ANTD / No PNTD Age^2 ^b^ | -0.096 | -0.107 | -0.085 | 0.006 | <.001 |
| No ANTD / No PNTD Age^3 ^c^ | -0.005 | -0.006 | -0.004 | 0.0005 | <.001 |
| No ANTD / No PNTD Age^4 ^d^ | 0.002 | 0.002 | 0.002 | 0.0001 | <.001 |
| Yes ANTD / No PNTD Intercept | -0.265 | -1.219 | 0.688 | 0.486 | 0.585 |
| Yes ANTD / No PNTD Age ^a^ | 0.117 | -0.121 | 0.354 | 0.121 | 0.336 |
| Yes ANTD / No PNTD Age^2 ^b^ | 0.042 | -0.034 | 0.118 | 0.039 | 0.278 |
| Yes ANTD / No PNTD Age^3 ^c^ | -0.004 | -0.010 | 0.002 | 0.003 | 0.228 |
| Yes ANTD / No PNTD Age^4 ^d^ | 0.000 | -0.002 | 0.001 | 0.001 | 0.687 |
| No ANTD / Yes PNTD Intercept | 0.164 | -0.831 | 1.158 | 0.507 | 0.747 |
| No ANTD / Yes PNTD Age ^a^ | -0.049 | -0.305 | 0.207 | 0.131 | 0.706 |
| No ANTD / Yes PNTD Age^2 ^b^ | 0.007 | -0.072 | 0.086 | 0.040 | 0.862 |
| No ANTD / Yes PNTD Age^3 ^c^ | 0.003 | -0.004 | 0.009 | 0.003 | 0.429 |
| No ANTD / Yes PNTD Age^4 ^d^ | -0.0001 | -0.002 | 0.001 | 0.001 | 0.865 |
| Yes ANTD / Yes PNTD Intercept | 1.790 | 0.414 | 3.166 | 0.702 | 0.011 |
| Yes ANTD / Yes PNTD Age ^a^ | 0.118 | -0.217 | 0.453 | 0.171 | 0.491 |
| Yes ANTD / Yes PNTD Age^2 ^b^ | -0.033 | -0.141 | 0.075 | 0.055 | 0.553 |
| Yes ANTD / Yes PNTD Age^3 ^c^ | 0.000 | -0.009 | 0.008 | 0.004 | 0.911 |
| Yes ANTD / Yes PNTD Age^4 ^d^ | 0.001 | -0.001 | 0.003 | 0.001 | 0.528 |

**Table S6. Paternal unadjusted trajectories of offspring depressive symptoms (n=5,584)**

Note: The No ANTD/No PNTD variable is the baseline (reference) group. There are four groups. Each group has an intercept and four terms resulting in four trajectories per group. The different terms (a-d) for each group are further parameters to account for the non-linearity of the trajectories as seen by the fact that the slope is steeper at different ages. Here we present the original regression coefficients and their 95%Cis, standard errors and p values for each group. Intercepts are centred to age 16, the mean age of all assessments.

Abbreviations: ANTD- antenatal depression; PNTD-postnatal depression; ^a^- linear slope, ^b^- quadratic slope, ^c^- cubic change in speed of slope, ^d^-quartic slope

| Table S7. Maternal adjusted trajectories of offspring depressive symptoms by sex (n=5,029) | | | | | | | | | | | | | |
| --- | --- | --- | --- | --- | --- | --- | --- | --- | --- | --- | --- | --- | --- |
|  | Parameter | Estimate | 95% CIs | | Std. Err. | P-Value |  | Parameter | Estimate | 95% CIs | | Std. Err. | P-Value |
| Males | No ANTD / No PNTD Intercept | 4.485 | 4.234 | 4.736 | 0.128 | <.001 | **Females** | No ANTD / No PNTD Intercept | 6.688 | 6.455 | 6.920 | 0.153 | <.001 |
|  | No ANTD / No PNTD Age ^a^ | 0.270 | 0.213 | 0.328 | 0.029 | <.001 |  | No ANTD / No PNTD Age ^a^ | 0.362 | 0.311 | 0.413 | 0.039 | 0.02 |
|  | No ANTD / No PNTD Age^2 ^b^ | -0.036 | -0.055 | -0.017 | 0.010 | <.001 |  | No ANTD / No PNTD Age^2 ^b^ | -0.126 | -0.141 | -0.110 | 0.012 | <.001 |
|  | No ANTD / No PNTD Age^3 ^c^ | -0.005 | -0.006 | -0.003 | 0.001 | <.001 |  | No ANTD / No PNTD Age^3 ^c^ | -0.004 | -0.006 | -0.003 | 0.001 | 0.732 |
|  | No ANTD / No PNTD Age^4 ^d^ | 0.001 | 0.001 | 0.001 | 0.0002 | <.001 |  | No ANTD / No PNTD Age^4 ^d^ | 0.002 | 0.002 | 0.002 | 0.0002 | <.001 |
|  | Yes ANTD / No PNTD Intercept | 6.129 | 5.283 | 6.975 | 0.442 | <.001 |  | Yes ANTD / No PNTD Intercept | 7.920 | 7.215 | 8.625 | 0.371 | <.001 |
|  | Yes ANTD / No PNTD Age ^a^ | 0.389 | 0.173 | 0.605 | 0.114 | 0.297 |  | Yes ANTD / No PNTD Age ^a^ | 0.405 | 0.224 | 0.586 | 0.097 | 0.164 |
|  | Yes ANTD / No PNTD Age^2 ^b^ | -0.132 | -0.205 | -0.060 | 0.038 | 0.012 |  | Yes ANTD / No PNTD Age^2 ^b^ | -0.136 | -0.190 | -0.082 | 0.029 | 0.001 |
|  | Yes ANTD / No PNTD Age^3 ^c^ | -0.008 | -0.014 | -0.003 | 0.003 | 0.204 |  | Yes ANTD / No PNTD Age^3 ^c^ | -0.003 | -0.008 | 0.002 | 0.003 | 0.536 |
|  | Yes ANTD / No PNTD Age^4 ^d^ | 0.003 | 0.0010 | 0.004 | 0.001 | 0.011 |  | Yes ANTD / No PNTD Age^4 ^d^ | 0.002 | 0.0010 | 0.003 | 0.001 | 0.04 |
|  | No ANTD / Yes PNTD Intercept | 4.748 | 3.765 | 5.731 | 0.510 | 0.606 |  | No ANTD / Yes PNTD Intercept | 7.840 | 6.991 | 8.689 | 0.443 | <.001 |
|  | No ANTD / Yes PNTD Age ^a^ | 0.376 | 0.123 | 0.630 | 0.133 | 0.424 |  | No ANTD / Yes PNTD Age ^a^ | 0.577 | 0.360 | 0.795 | 0.115 | 0.008 |
|  | No ANTD / Yes PNTD Age^2 ^b^ | -0.40 | -0.123 | 0.042 | 0.043 | 0.918 |  | No ANTD / Yes PNTD Age^2 ^b^ | -0.123 | -0.188 | -0.058 | 0.034 | 0.011 |
|  | No ANTD / Yes PNTD Age^3 ^c^ | -0.006 | -0.013 | 0.0001 | 0.003 | 0.636 |  | No ANTD / Yes PNTD Age^3 ^c^ | -0.006 | -0.012 | -0.0001 | 0.003 | 0.6 |
|  | No AND / Yes PNTD Age^4 ^d^ | 0.0010 | -0.001 | 0.003 | 0.001 | 0.877 |  | No ANTD / Yes PNTD Age^4 ^d^ | 0.002 | 0.0010 | 0.003 | 0.001 | 0.134 |
|  | Yes ANTD / Yes PNTD Intercept | 6.211 | 4.904 | 7.518 | 0.672 | 0.01 |  | No ANTD / Yes PNTD Intercept | 8.482 | 7.348 | 9.616 | 0.584 | <.001 |
|  | Yes ANTD / Yes PNTD Age ^a^ | 0.749 | 0.409 | 1.088 | 0.176 | 0.007 |  | No ANTD / Yes PNTD Age ^a^ | 0.605 | 0.300 | 0.910 | 0.159 | 0.035 |
|  | Yes ANTD / Yes PNTD Age^2 ^b^ | -0.114 | -0.225 | -0.003 | 0.057 | 0.172 |  | No ANTD / Yes PNTD Age^2 ^b^ | -0.136 | -0.229 | -0.042 | 0.049 | 0.041 |
|  | Yes ANTD / Yes PNTD Age^3 ^c^ | -0.018 | -0.027 | -0.009 | 0.004 | 0.002 |  | No ANTD / Yes PNTD Age^3 ^c^ | -0.007 | -0.015 | 0.001 | 0.004 | 0.581 |
|  | Yes ANTD / Yes PNTD Age^4 ^d^ | 0.003 | 0.0001 | 0.005 | 0.001 | 0.145 |  | Yes ANTD / Yes PNTD Age^4 ^d^ | 0.003 | 0.00100 | 0.005 | 0.001 | 0.051 |

Note. The No ANTD/No PNTD variable is the baseline (reference) group. There are four groups. Each group has an intercept and four terms resulting in four trajectories per group. The different terms (a-d) for each group are further parameters to account for the non-linearity of the trajectories as seen by the fact that the slope is steeper at different ages. The intercept for each group was determined by manually adding the baseline intercept for No ANTD/No PNTD to the intercept of the group being compared. Similarly, the rate of change for each subsequent group was determined by adding the rate of change for the baseline group to the rate of change of the group being compared. For ease of interpretation we are presenting this data already combined. The standard error and p value represent the difference between the baseline trajectory and the additional parameter. This analysis was adjusted for sex, paternal depression, maternal education at birth, parity and maternal smoking in pregnancy. Intercepts are centred to age 16, the mean age of all assessments.

Abbreviations: ANTD- antenatal depression; PNTD-postnatal depression; ^a^- linear slope, ^b^- quadratic slope, ^c^- cubic change in speed of slope, ^d^-quartic slope

**Figure S2 – Maternal depression and offspring trajectories by sex**

***Abbreviations: ANTD- antenatal depression; PNTD-postnatal depression; SMFQ- short mood and feelings questionnaire**

| Table S8. Paternal adjusted trajectories of offspring depressive symptoms by sex (n=4,534) | | | | | | | | | | | | | |
| --- | --- | --- | --- | --- | --- | --- | --- | --- | --- | --- | --- | --- | --- |
|  | Parameter | Estimate | 95% CIs | | Std. Err. | P-Value |  | Parameter | Estimate | 95% CIs | | Std. Err. | P-Value |
| Males | No ANTD / No PNTD Intercept | 4.528 | 4.260 | 4.796 | 0.137 | <.001 | Females | No ANTD / No PNTD Intercept | 6.635 | 6.387 | 6.882 | 0.151 | <.001 |
|  | No ANTD / No PNTD Age ^a^ | 0.299 | 0.242 | 0.357 | 0.029 | <.001 |  | No ANTD / No PNTD Age ^a^ | 0.374 | 0.324 | 0.425 | 0.039 | 0.055 |
|  | No ANTD / No PNTD Age^2 ^b^ | -0.044 | -0.063 | -0.026 | 0.010 | <.001 |  | No ANTD / No PNTD Age^2 ^b^ | -0.122 | -0.137 | -0.107 | 0.012 | <.001 |
|  | No ANTD / No PNTD Age^3 ^c^ | -0.005 | -0.007 | -0.004 | 0.001 | <.001 |  | No ANTD / No PNTD Age^3 ^c^ | -0.004 | -0.006 | -0.003 | 0.001 | 0.248 |
|  | No ANTD / No PNTD Age^4 ^d^ | 0.001 | 0.001 | 0.002 | 0.0002 | <.001 |  | No ANTD / No PNTD Age^4 ^d^ | 0.002 | 0.002 | 0.002 | 0.0002 | 0.001 |
|  | Yes ANTD / No PNTD Intercept | 3.365 | 1.900 | 4.830 | 0.751 | 0.122 |  | Yes ANTD / No PNTD Intercept | 7.648 | 5.929 | 9.367 | 0.880 | <.001 |
|  | Yes ANTD / No PNTD Age ^a^ | 0.189 | -0.205 | 0.583 | 0.203 | 0.586 |  | Yes ANTD / No PNTD Age ^a^ | 0.356 | -0.074 | 0.787 | 0.222 | 0.797 |
|  | Yes ANTD / No PNTD Age^2 ^b^ | 0.041 | -0.084 | 0.166 | 0.064 | 0.186 |  | Yes ANTD / No PNTD Age^2 ^b^ | -0.174 | -0.316 | -0.032 | 0.073 | 0.076 |
|  | Yes ANTD / No PNTD Age^3 ^c^ | -0.003 | -0.012 | 0.007 | 0.005 | 0.574 |  | Yes ANTD / No PNTD Age^3 ^c^ | -0.002 | -0.013 | 0.010 | 0.006 | 0.544 |
|  | Yes ANTD / No PNTD Age^4 ^d^ | -0.0001 | -0.003 | 0.002 | 0.001 | 0.341 |  | Yes ANTD / No PNTD Age^4 ^d^ | 0.003 | -0.0001 | 0.006 | 0.001 | 0.240 |
|  | No ANTD / Yes PNTD Intercept | 4.474 | 2.933 | 6.015 | 0.789 | 0.946 |  | No ANTD / Yes PNTD Intercept | 6.619 | 5.168 | 8.070 | 0.745 | 0.005 |
|  | No ANTD / Yes PNTD Age ^a^ | 0.245 | -0.165 | 0.654 | 0.211 | 0.797 |  | No ANTD / Yes PNTD Age ^a^ | 0.292 | -0.103 | 0.688 | 0.204 | 0.973 |
|  | No ANTD / Yes PNTD Age^2 ^b^ | -0.05 | -0.188 | 0.087 | 0.071 | 0.931 |  | No ANTD / Yes PNTD Age^2 ^b^ | -0.071 | -0.184 | 0.041 | 0.058 | 0.644 |
|  | No ANTD / Yes PNTD Age^3 ^c^ | -0.003 | -0.013 | 0.0080 | 0.005 | 0.602 |  | No ANTD / Yes PNTD Age^3 ^c^ | -0.001 | -0.012 | 0.010 | 0.005 | 0.407 |
|  | No ANTD / Yes PNTD Age^4 ^d^ | 0.001 | -0.002 | 0.004 | 0.001 | 0.957 |  | No ANTD / Yes PNTD Age^4 ^d^ | 0.001 | -0.0020 | 0.003 | 0.001 | 0.785 |
|  | Yes ANTD / Yes PNTD Intercept | 4.703 | 2.465 | 6.942 | 1.144 | 0.878 |  | Yes ANTD / Yes PNTD Intercept | 9.019 | 6.992 | 11.047 | 1.038 | <.001 |
|  | Yes ANTD / Yes PNTD Age ^a^ | 0.287 | -0.244 | 0.818 | 0.272 | 0.964 |  | Yes ANTD / Yes PNTD Age ^a^ | 0.700 | 0.150 | 1.251 | 0.282 | 0.156 |
|  | Yes ANTD / Yes PNTD Age^2 ^b^ | -0.055 | -0.253 | 0.143 | 0.101 | 0.920 |  | Yes ANTD / Yes PNTD Age^2 ^b^ | -0.197 | -0.352 | -0.042 | 0.080 | 0.055 |
|  | Yes ANTD / Yes PNTD Age^3 ^c^ | -0.006 | -0.019 | 0.008 | 0.007 | 0.982 |  | Yes ANTD / Yes PNTD Age^3 ^c^ | -0.009 | -0.023 | 0.006 | 0.008 | 0.684 |
|  | Yes ANTD / Yes PNTD Age^4 ^d^ | 0.001 | -0.0030 | 0.005 | 0.002 | 0.974 |  | Yes ANTD / Yes PNTD Age^4 ^d^ | 0.004 | 0.0001 | 0.007 | 0.002 | 0.122 |

Note: The No ANTD/No PNTD variable is the baseline (reference) group. There are four groups. Each group has an intercept and four terms resulting in four trajectories per group. The different terms (a-d) for each group are further parameters to account for the non-linearity of the trajectories as seen by the fact that the slope is steeper at different ages. The intercept for each group was determined by manually adding the baseline intercept for No ANTD/No PNTD to the intercept of the group being compared. Similarly the rate of change for each subsequent group was determined by adding the rate of change for the baseline group to the rate of change of the group being compared. For ease of interpretation we are presenting this data already combined. The standard error and p value represent the difference between the baseline trajectory and the additional parameter. This analysis was adjusted for sex, maternal depression, paternal education at birth, parity and paternal smoking in pregnancy. Intercepts are centred to age 16, the mean age of all assessments.

Abbreviations: ANTD- antenatal depression; PNTD-postnatal depression; ^a^- linear slope, ^b^- quadratic slope, ^c^- cubic change in speed of slope, ^d^-quartic slope

**Figure S3 – Paternal depression and offspring trajectories by sex**

***Abbreviations: ANTD- antenatal depression; PNTD-postnatal depression; SMFQ- short mood and feelings questionnaire**

|  | Table S9. Different intercept scores for maternal ANTD/PNTD groups and offspring trajectories of DS | | | | |
| --- | --- | --- | --- | --- | --- |
| Intercept scores (95% CIs) of DS trajectories at different ages | | | | |  |
|  | 12 | 16 | 20 | 24 |  |
| No ANTD / No PNTD | 3.19 (3.01, 3.37) | 5.15 (4.95, 5.36) | 5.47 (5.24, 5.70) | 5.92 (5.64, 6.20) |  |
| Yes ANTD / No PNTD | 3.73 (3.31, 4.16) | 6.52 (5.98, 7.06) | 6.75 (6.09, 7.42) | 7.82 (6.96, 8.68) |  |
| No ANTD / Yes PNTD | 3.35 (2.86, 3.85) | 5.84 (5.20, 6.47) | 6.81 (6.03, 7.59) | 7.34 (6.31, 8.36) |  |
| Yes ANTD / Yes PNTD | 3.73 (3.07, 4.38) | 6.67 (5.82, 7.51) | 7.90 (6.82, 8.98) | 8.81 (7.36, 10.26) |  |

Note: Adjusted for sex, paternal depression, maternal education at birth, parity and maternal smoking during pregnancy. Abbreviations: ANTD- antenatal depression; PNTD-postnatal depression, DS- depressive symptoms. The total N for this analysis is 5,029. The numbers in each group are: No ANTD / No PNTD =4,264, Yes ANTD / No PNTD = 365, No ANTD / Yes PNTD = 253, Yes ANTD / Yes PNTD =147

|  | Table S10. Different intercept scores for paternal ANTD/PNTD groups and offspring trajectories of DS | | | | |
| --- | --- | --- | --- | --- | --- |
| Intercept scores (95% CIs) of DS trajectories at different ages | | | | |  |
|  | 12 | 16 | 20 | 24 |  |
| No ANTD / No PNTD | 3.14 (2.94, 3.35) | 5.19 (4.96, 5.42) | 5.56 (5.31, 5.81) | 5.96 (5.67, 6.25) |  |
| Yes ANTD / No PNTD | 3.47 (2.63, 4.32) | 4.80 (3.70, 5.89) | 5.36 (3.99, 6.74) | 6.82 (4.85, 8.78) |  |
| No ANTD / Yes PNTD | 3.33 (2.49, 4.18) | 5.10 (4.06, 6.14) | 5.64 (4.35, 6.92) | 6.18 (4.37, 7.99) |  |
| Yes ANTD / Yes PNTD | 3.41 (2.30, 4.52) | 6.45 (4.99, 7.91) | 7.08 (5.34, 8.82) | 8.59 (6.51, 10.68) |  |

Note: Adjusted for sex, maternal depression, paternal education at birth, parity and paternal smoking during pregnancy. Abbreviations: AND- antenatal depression; PNTD-postnatal depression; DS- depressive symptoms. The total N for this analysis is 4,534. The numbers in each group are: No ANTD / No PNTD =4,317, Yes ANTD / No PNTD = 80, No ANTD / Yes PNTD = 87, Yes ANTD / Yes PNTD = 50

**Figure S4. Comparisons between linear (A), quadratic (B), cubic (C) and quartic (D) models
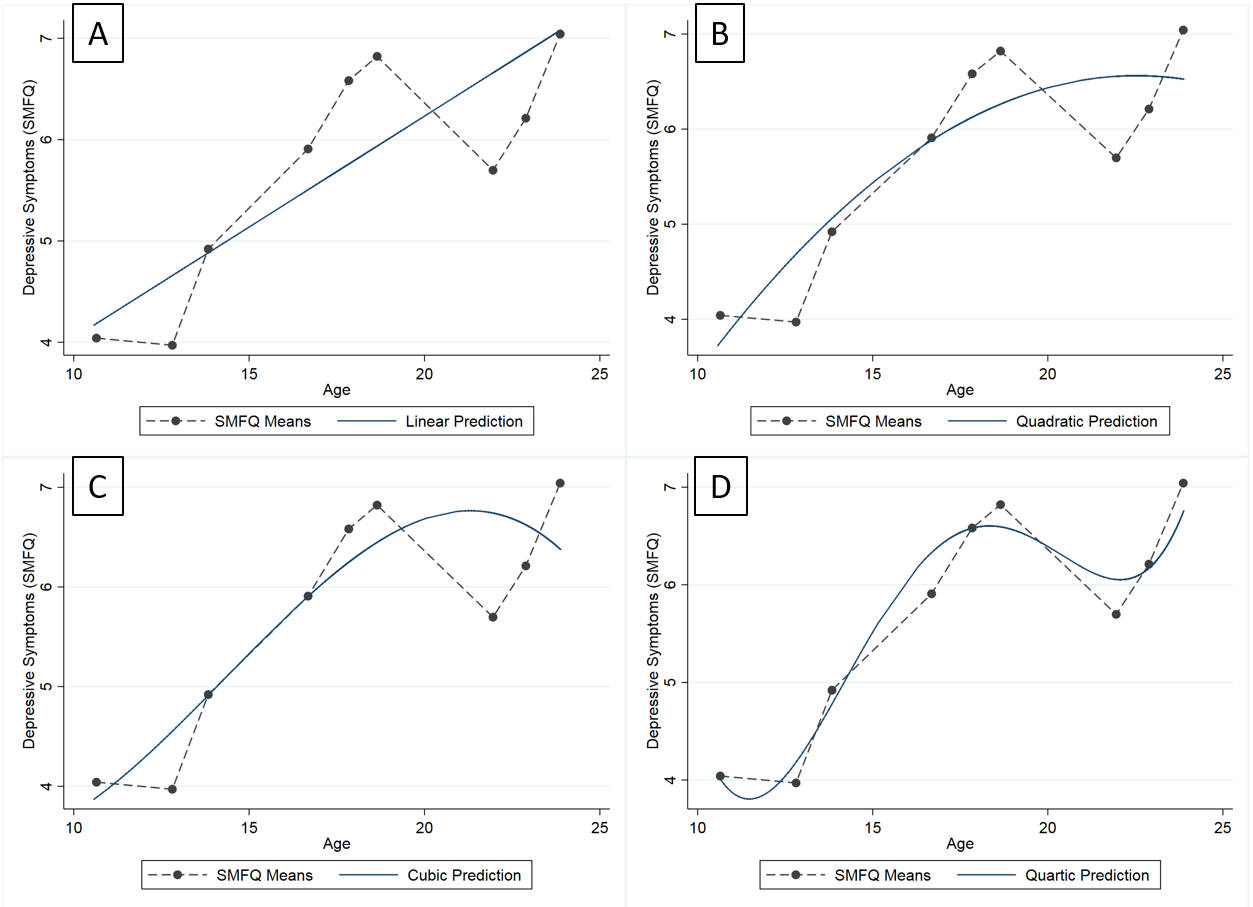
**

| Table S11. Comparisons between polynomial models with SMFQ data (n=9,399). | | | |
| --- | --- | --- | --- |
| Model | **Deviance** | **AIC** | **BIC** |
| Linear Polynomial Model | 254854.65 | 254866.7 | 254918.8 |
| Quadratic Polynomial Model | 253471.09 | 253491.1 | 253578 |
| Cubic Polynomial Model | 253076.68 | 253106.7 | 253237 |
| Quartic Polynomial Model | 252218.3 | 252260.3 | 252442.8 |

Model fit by the four polynomial models. SMFQ: short mood and feelings questionnaire; AIC: Akaike information criterion;

BIC: Bayesian information criterion

| Table S12. Comparisons between polynomial models with maternal depression and all confounders (n=5,029). | | | |
| --- | --- | --- | --- |
| Model | **Deviance** | **AIC** | **BIC** |
| Linear Polynomial Model | 147798.27 | 147810.3 | 147859.2 |
| Quadratic Polynomial Model | 147006.06 | 147026.1 | 147107.6 |
| Cubic Polynomial Model | 146747.3 | 146777.3 | 146899.6 |
| Quartic Polynomial Model | **146286.59** | **146328.6** | **146499.8** |

Model fit by the four polynomial models. SMFQ: short mood and feelings questionnaire; AIC: Akaike information criterion;

BIC: Bayesian information criterion

| Table S13. Comparisons between polynomial models with paternal depression and all confounders (n=4,534). | | | |
| --- | --- | --- | --- |
| Model | **Deviance** | **AIC** | **BIC** |
| Linear Polynomial Model | 134214.22 | 134226.2 | 134274.2 |
| Quadratic Polynomial Model | 133461.48 | 133481.5 | 133562.1 |
| Cubic Polynomial Model | 133210.13 | 133240.1 | 133361 |
| Quartic Polynomial Model | **132805.7** | **132847.7** | **133017** |

Model fit by the four polynomial models. SMFQ: short mood and feelings questionnaire; AIC: Akaike information criterion;

BIC: Bayesian information criterion

1. We also compared intercepts at different ages in S9 and S10. [↑](#footnote-ref-1)
